# Detection ranges of forest bird vocalisations: guidelines for passive acoustic monitoring

**DOI:** 10.1038/s41598-024-51297-z

**Published:** 2024-01-09

**Authors:** Dominika Winiarska, Paweł Szymański, Tomasz S. Osiejuk

**Affiliations:** Department of Behavioural Ecology, Institute of Environmental Biology, Faculty of Biology, Adam Mickiewicz University, Poznań, Poland

**Keywords:** Ecology, Zoology

## Abstract

Passive acoustic monitoring has proven to have many advantages for monitoring efforts and research activities. However, there are considerations to be taken into account regarding the placement of autonomous sound recorders. Detection ranges differ among species and in response to variable conditions such as weather or the location of vocalising animals. It is thus important to the success of a research project to understand, with a certain degree of confidence, the distances at which birds might be detected. In two types of forests in Poland, we played back the vocalisations of 31 species of European forest birds exemplifying different singing characteristics. Based on recordings obtained along a 500-m transect, we estimated the probability of detection and maximum detection distance of each vocalisation. We broadcasted the recording at three heights of singing and repeated playbacks three times during the breeding season to evaluate the effect of vegetation growth. Our results revealed that environmental and meteorological factors had a significant influence on both detection probability and maximum detection distances. This work provides comprehensive measurements of detection distance for 31 bird species and can be used to plan passive acoustic monitoring research in Europe, taking into account species traits and individual characteristics of the study area.

## Introduction

Estimations of population abundance or the composition of species assemblages at a given spatial and temporal scale are invaluable for ecological research but also serve as the starting point for determining the conservation status of populations. Regardless of the purpose for which such data are collected, abundance estimates should reflect, to the greatest possible extent, the actual status of populations. Until recently, the most reliable way to accomplish this was using human experts, but rapid technological development has provided new tools of automated data collection that in many areas can effectively replace traditional methods of monitoring populations^1^.

Passive acoustic monitoring (PAM) is based on the deployment of autonomous recording units (ARUs) in the field to record soundscapes. PAM has been shown to be a reliable tool for a wide range of research topics and vocalising taxa^2^. Compared to traditional monitoring methods, ARUs can be easily deployed, even in remote areas, and can operate without human attention for long periods of time. They are fully programmable at various temporal scales and are cost-effective. Most importantly, however, ARUs can collect data in a fully reproducible manner even over very long periods and large spatial scales^3^. Furthermore, acoustic data can be stored for future comparisons^4^.

While PAM has many advantages, a few shortcomings remain. To be directly comparable among different projects, PAM recordings must be collected in a standardised way. Despite exponential growth in the use of PAM in recent years, there are very few practical guides on how to deploy devices to accurately investigate areas of interest. The available suggestions focus on detectability or device settings when recording different species (see^5–8^) and some recommend measuring sound detection spaces separately in each habitat^9^. In general, ARUs are deployed based on the subject and aims of the study or monitoring programme at hand, and recommendations regarding the sampling of a given species/assemblage also tend to reflect these different priorities. For this reason, acoustic data from different projects are often not suitable for comparisons as, for instance, they do not contain information about species’ detection probabilities. The biggest problem seems to be the unknown detection distance, which is species-specific and varies depending on habitat. In addition, issues related to ARU spacing have been poorly investigated, such as data duplication or gaps due to ARUs being too close or too far apart, respectively. Signal detection is affected by a variety of biotic (both interspecific and intraspecific) and abiotic factors that influence sound propagation^10^. Furthermore, the technical parameters of the ARUs’ microphones only give information on the potential range to be covered, which can be modified by the choice of settings even within a single device.

One of the reasons for the lack of precise guidelines is insufficient knowledge of the distances from which ARUs detect different animals, including birds^11^. Knowledge about maximum detection distances gives some basic information about the possibility of detecting or missing a particular species and is crucial for planning the spatial distribution of ARUs to ensure that they are independent, i.e., record only different individuals of the monitored species. There have been various proposals on factors to consider when determining the range of an ARU, of which one is the effective detection radius, i.e., the distance beyond which as many vocalisations are undetected as detected^12^. To determine this, however, we need more data on the probability of detecting various species in different habitats, with more accurate distances for different animals or species. With this information, it is possible to provide more precise guidelines on how to deploy equipment to record particular species or animal groups of interest.

In this study, we conducted an extensive range-testing experiment to evaluate the effect of forest habitats, time of season, and height of sound source on the detection probability of acoustic signals of selected European temperate-forest bird species. Our main objective was to provide practical advice for the planning and interpretation of avian bioacoustics monitoring in temperate forest habitats. We selected a set of bird species with differentiated song characteristics—song duration, frequency characteristics, and amplitude—to evaluate how different vocalisation parameters affect detection ranges. We also analysed how environmental factors affect detection distances to make our results more generally applicable and promote their use in monitoring different forest species.

## Materials and methods

### Study site

**Figure 1:**
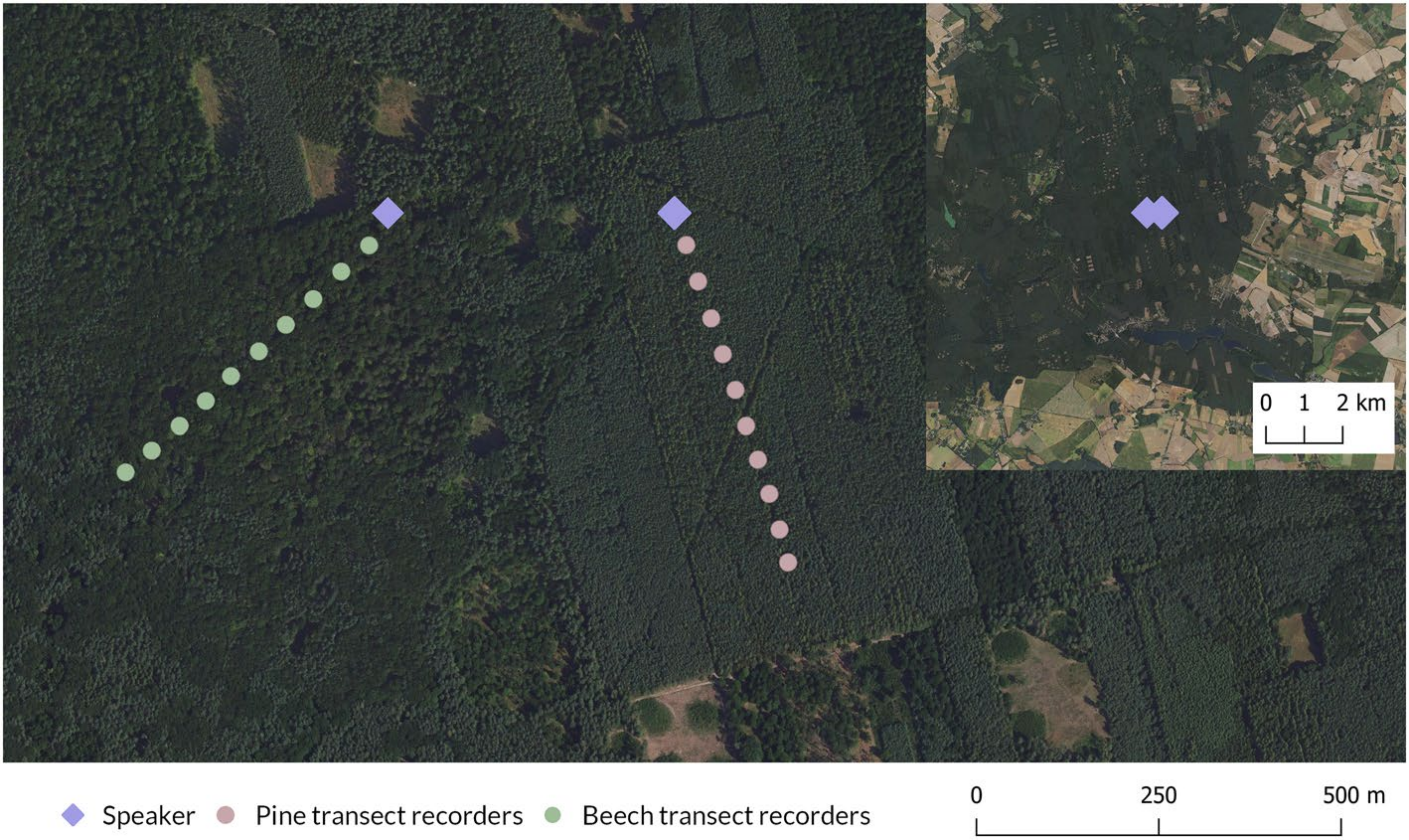
Aerial map of the study area. Ten ARUs were placed 50 m apart along two 500-m transects at 4 m height. The speaker at the beginning of each transect was mounted at 3, 6, or 9 m height. The larger scale inset shows a lack of any urban agglomeration close to the site. The map was made using QGIS software and maps provided by the Geoportal service^14,15^.

The experiments were carried out in the Zielonka forest near the city of Poznań, in western Poland (N 52.545500, E 17.150639), which is characterised by different types of temperate woodlands. Two transects were designed, separated by ca. 400 m at their beginnings and ca. 900 m at their ends (Fig. 1). The first site was a coniferous forest, mainly consisting of the pine tree *Pinus sylvestris*. The other was a nearby deciduous forest dominated by common beech *Fagus sylvatica* and sessile oak *Quercus petrea.* The average height of trees in the coniferous forest was ca. 22 m, with a 4-m canopy diameter; the mean stand age was 48 years (range 44–53 years). In the deciduous forest, trees were about 25 m in height with a mean canopy diameter of 9 m, and were more differentiated in age (mean 116 years, range 12–257). The pine forest appeared slightly denser due to its smaller canopy size, though both sites are marked as moderately dense forests^13^. In the beech forest, the leaves gradually developed throughout the research period. The experiments were conducted in 2021. In April the trees had small buds; in May they burst into small leaves that fully developed in June. The coniferous forest needles did not exhibit changes throughout the season, but the underbrush, consisting of taller grasses and ferns, developed in June. Instead, the deciduous forest featured a layer of leaves from the previous year that did not change much throughout the season. The topology of both sites was flat to avoid any influence of the terrain structure on sound propagation. Both sites were located ~ 3 km from the nearest public and tarmac road, so there were no anthropogenic sounds.

### Design of transmission experiment

Along each transect, we placed 10 ARUs (Song Meter SM4 with two built-in omnidirectional microphones, Wildlife Acoustics, Maynard, MA, USA), one every 50 m, so that they were located between 50 and 500 m from a loudspeaker (position measured with GPS Garmin GPSMAP 65 s with averaged accuracy ± 3 m). We used built-in omnidirectional microphones with sensitivity − 35 ± 4 dB and signal-to-noise ratio 80 dB, calibrated using sound level calibrator (VOLTCRAFT SLC-100). Each ARU was randomly picked and mounted on a tree trunk at a height of 4 m. Sound files were saved as uncompressed PCM.wav files with a 48 kHz sampling rate and 16-bit resolution. The loudspeaker (JBL XTREME 3, Harman, CA, USA; output power 25W RMS, frequency response range 53.5 Hz–20 kHz) used for broadcasting the recordings was placed at a height of 3, 6, or 9 m at the starting points of both transects. The choice of height for each subsequent broadcast sequence was pseudo-random to prevent, for example, playback from the highest location always occurring earliest in the morning. Broadcasting on the two transects was performed on two consecutive days at the beginning of each month, from April to June, starting one hour after sunrise to avoid significant competition from the dawn chorus. We also measured temperature, humidity, and wind speed during the beginning and end of each broadcast using a Benetech GT8907 (Shenzhen, China) weather meter. During all experimental days, the weather conditions were favourable with no rain and no strong (> 1 m/s) wind. Meteorological data is available in Supplementary Table 1.

### Bird vocalisations used for playback

We wanted to examine a wide range of vocalisation characteristics that can affect the probability of detection, such as frequency, amplitude, and duration. For this, we selected songs and territorial calls of 31 European forest bird species (Table 1). The playback began with a series of three pure tones at a frequency of 1000 Hz generated using Avisoft SASLab Pro (version 5.2.14, R. Specht, Berlin, Germany), so that we could easily find the beginning of each playback in the recordings. After this initial sequence, we broadcasted one minute of playback for each species, featuring 2–6 types of vocalisations produced at a species-specific rate. The number of distinctive vocalisations depended on the number of recordings available, and the number of repetitions within the minute-long period was determined by the length of the individual vocalisations. After the songs or territorial calls of each species, we played three pure tones at a frequency of 1000 Hz to mark the end of each species’ playback section. Each pure tone lasted 0.5 s and was separated from the others by ca. 1.5 s of silence. The total duration of the broadcast series was around 45 min.

**Table 1:** List of species used for playback in sound propagation experiments.

Species	Body mass (g)	Minimum frequency (Hz)	Maximum frequency (Hz)	Bandwidth (Hz)	Peak frequency (Hz)	Amplitude measured at 1 m (dBA)	Group
*Accipiter nissus*	151	1990	4361	2438	3402	82.5	Medium
*Aegithalos caudatus*	8	1901	8856	6955	4436	79.3	Small
*Anthus trivialis*	27	1923	8073	6195	4350	86.0	Small
*Certhia brachydactyla*	9	4428	7469	3198	5082	85.8	Small
*Certhia familiaris*	9	2751	8789	6038	6891	81.7	Small
*Columba oenas*	334	127	1297	1171	431	92.2	Large
*Columba palumbus*	487	158	1946	1787	517	90.2	Large
*Cuculus canorus*	124	268	984	872	689	91.8	Large
*Cyanistes caeruleus*	11	4115	8789	5144	6202	85.2	Small
*Dendrocopos major*	84	716	6105	5390	3618	84.8	Medium
*Dryocopus martius*	310	1051	2281	1230	1895	90.9	Medium
*Erithacus rubecula*	20	1968	9012	7492	3790	84.6	Small
*Ficedula hypoleuca*	16	1901	9035	7134	4177	86.1	Small
*Ficedula parva*	10	2438	7425	6038	4134	87.2	Small
*Fringilla coelebs*	23	1565	8140	6575	3747	85.0	Small
*Lophophanes cristatus*	13	2572	7134	5479	5513	82.6	Small
*Loxia curvirostra*	35	1834	10,175	8341	4221	84.9	Small
*Lullula arborea*	29	2013	5412	3399	3445	86.4	Small
*Luscinia megarhynchos*	28	1029	12,712	11,684	3273	88.3	Small
*Oriolus oriolus*	72	738	4652	6060	1981	88.0	Medium
*Parus major*	17	3176	6821	4808	4221	91.1	Small
*Phylloscopus colybita*	9	3109	7626	5770	4350	81.9	Small
*Phylloscopus sibilatrix*	11	3041	9079	6150	4048	82.5	Small
*Phylloscopus trochilus*	10	2125	6798	5546	3919	82.8	Small
*Poecile palustris*	12	2706	7402	5322	4350	81.7	Small
*Prunella modularis*	20	2348	8386	6374	4651	81.6	Small
*Regulus regulus*	6	4830	8297	6239	7020	80.4	Small
*Sylvia atricapilla*	20	1498	7402	7581	3058	86.7	Small
*Troglodytes troglodytes*	9	3131	8587	6083	5125	90.9	Small
*Turdus merula*	95	1208	8028	6821	2412	85.9	Medium
*Turdus philomelos*	79	1633	6620	5054	2929	86.8	Medium

Body mass is the mean weight of a male^16^ and was used to divide species into groups, using principal component analysis of body mass relative to peak frequency. Acoustic parameters represent the vocalisations used for playback.

Recordings were obtained mostly from The Bird Songs of Europe, North Africa, and the Middle East by Schulze and Dingler^17^. All recordings were first inspected for quality and filtered up to 500 Hz outside the band of the focal species if possible. The amplitudes of the chosen signals were modified in Avisoft SAS Lab Pro to match the real amplitude at which the species sings or calls (based on literature data, our own measurements, or similar species data). As a final check of the amplitude level, we played signals with a fixed setup of the speaker and recorder, and we measured the amplitude of the reproduced sounds. The speaker and decibel meter were placed 2 m from each other (to avoid taking measurements in the near field) on tripods 1.6-m high. All song sequences were reproduced three times from the speaker, and the maximum amplitude of each vocalisation was noted. Measurements were based on fast-time A-weighting using a CHY 650 Sound Level Meter (Ningbo, China). Measurements were taken outdoors in quiet conditions (ambient noise < 42 dB SPL). The average values for each species are presented in Table 1; they are recalculated and shown as the amplitude SPL dBA at a 1-m distance from the source.

Using principal component analysis, we divided all species into groups based on their peak frequency relative to body mass, in order to compare species with similar characteristics with each other and within groups. The body mass used was the mean weight for the male of each species^16^. The groups included large birds (with a peak frequency of 431–689 Hz), medium (1895–3618 Hz), and small ones (3058–7020 Hz). All parameters are presented in Table 1.

To inform recommendations on the minimum distances between automatic recording devices, we compared the detection distances of individual species with the average sizes of their territories. As reference values for the latter, we adopted the sizes of the territories found in a natural lowland forest, the Bialowieza Forest^18^. The values were converted from average densities. We used data from a lime-hornbeam forest to compare with our deciduous transect and a pine-bilberry coniferous forest for the coniferous one.

### Acoustic data processing

In total, we analysed 5580 sound files, each a 1-min sample of vocalisations of 1 of 31 species, re-recorded at a given distance, transect, height, and month. We excluded 117 recordings (2.1% of all samples) from the deciduous forest (7 in May, 110 in June) that were marked as errors due to recorder issues. We visually and audibly examined these sound files to determine if at least one of the re-recorded vocalisations could be either seen on the spectrogram or heard in each recording. If so, the detections were marked. This was done by one person (DW) with prior knowledge of which species’ vocalisation to search for in each recording. We used Raven Pro 1.6 (Cornell Lab of Ornithology, Ithaca) at default settings, including Hann window with 512 samples, Jet colour map, brightness, and contrast at 50. Settings were occasionally adjusted slightly to analyse difficult recordings.

To cross-check the initial verification, the recordings were also blindly analysed by PS and TO. We encrypted 310 randomly chosen recordings (5.56% of all samples), and all vocalisations seen in the spectrogram or heard in each recording were noted and identified. In the blind trial, the responses were 95% unanimous (295 samples).

### Statistical analysis

All statistical analyses were conducted in R^19^. First, to estimate the detection probability of each species’ vocalisation at each distance to the loudspeaker, we built a generalised linear mixed-effects model (GLMM) with binomial error distribution and probit link function using the ‘glmmTMB’ package^20^. Our response variable was a value indicating whether a given species’ vocalisation could be detected in a recording (1) or not (0). In the full model, we included the month, distance, the height of loudspeaker placement on an ordinal scale, and wind, humidity, temperature, time of day (minutes after sunrise), vocalisation peak frequency, vocalisation duration, species body mass and sound pressure level (SPL) as covariates scaled between 0 and 1. Transect type (deciduous vs coniferous) was included as fixed factor. Species was entered into the model as a random factor and distance was introduced as a random slope. Second, to answer the question of what factors affect the maximum distance at which a given species’ vocalisation can be detected, we built a GLMM with a truncated generalised Poisson distribution and log-link function to assess main effects. Our dependent variable was the maximum distance at which each species’ vocalisation was detected in each trial. Again, we included ordinally scaled month and the height of loudspeaker placement, with wind, humidity, temperature, time of day (minutes after sunrise), vocalisation peak frequency, vocalisation duration, species body mass and sound pressure level (SPL) as scaled covariates. Species identity was also entered into the model as a random factor. Variables were scaled to improve the model fit. We also tested the data for multicollinearity (both environmental variables and species characteristics), however this was not an issue. For both global models, we performed model selection using the ‘MuMIN’ package^21^. Among 4096 detection distance and 2045 maximum distance models, we identified the best model sets using Akaike’s information criterion (AIC^22^, Supplementary Table 2), using a threshold of ΔAIC = 2, and calculated the mean of the selected models to create an averaged model. Diagnostics of model residuals were performed with the help of the ‘DHARMa’ package^23^. To present the detection probability for each species we conducted logistic regression.

### Ethics statement

This study was carried out according to Polish law. We obtained all the necessary permissions to carry out the study: permission of the Regional Directorate of Environment Protection (no. WPN-II.6205.8.2021.JM) and permission of the Marshal of the Wielkopolska Region (no. DR-I.7131.1.2.2021).

## Results

### Detection ranges: general overview

We detected re-recorded species vocalisations in 1692 (30.3%) out of 5580 audio samples.

All of the best models indicated that the probability of detection of the vocalisations of the 31 species was most strongly affected by their body mass and SPL (Tables 2 and S2). As expected, the probability of detection was higher for larger species with low amplitude territorial calls or songs. Peak frequency and call duration did not have significant effect. Weather conditions also affected whether a vocalisation was detected, with temperature and humidity having a strong positive impact and wind a negative one. Furthermore, the probability of detection was lower at greater distances and for higher speaker placement, although the effect of height was minor. Interestingly, the probability of detecting a vocalisation was higher in the pine forest. Seasonal changes had a negative impact, whereas time of day did not significantly affect detectability.

**Table 2:** Averaged model results of detection probability and maximum distance used for the analysis.

Detection ~ distance + transect + height + month + body mass + peak frequency + SPL + call duration + time of day + temperature + humidity + wind + (1 + distance | species)					
	Estimate	Std. Error	Adjusted SE	z value	p value
Intercept	3.162	0.771	0.771	4.102	** < 0.001**
Distance	− 1.499	0.102	0.102	14.669	** < 0.001**
Transect beech	− 0.311	0.136	0.136	2.288	**0.022**
Height	− 0.132	0.048	0.048	2.740	**0.006**
Month	− 0.185	0.079	0.079	2.338	**0.019**
Body mass	4.404	0.949	0.949	4.640	** < 0.001**
Peak frequency	− 0.422	0.848	0.848	0.497	0.619
SPL	2.400	0.694	0.694	3.458	**0.001**
Call duration	0.264	0.538	0.538	0.490	0.624
Time of day	− 0.310	0.207	0.207	1.499	0.134
Temperature	1.308	0.273	0.273	4.795	** < 0.001**
Humidity	0.635	0.142	0.142	4.484	** < 0.001**
Wind	− 0.568	0.204	0.204	2.780	**0.005**

Max distance ~ transect + height + month + body mass + peak frequency + SPL + call duration + time of day + temperature + humidity + wind + (1 | species)					
	Estimate	Std. error	Adjusted SE	z value	p value
Intercept	− 2.468	1.261	1.264	1.953	0.051
Transect pine	0.112	0.028	0.029	3.940	** < 0.001**
Height	− 0.041	0.013	0.013	3.188	**0.001**
Month	− 0.047	0.020	0.020	2.397	**0.017**
Body mass	0.179	0.057	0.057	3.123	**0.002**
Peak frequency	− 0.355	0.147	0.148	2.404	**0.016**
SPL	3.286	1.187	1.189	2.763	**0.006**
Call duration	0.028	0.068	0.068	0.414	0.679
Time of day	0.114	0.047	0.047	2.400	**0.016**
Temperature	− 0.073	0.035	0.035	2.063	**0.039**
Humidity	0.520	0.129	0.130	4.011	** < 0.001**
Wind	− 0.011	0.033	0.033	0.330	0.742

Significant outcomes are bolded.

Similarly, SPL and humidity were also the variables with the greatest effect on the maximum detection distance. Maximum distance rose with increasing humidity. Longer-range detections were noted in the pine forest and at lower broadcasting height. Higher peak frequency shortened the distance, while larger and louder species were detectable at longer distances. The time of day was positively correlated with detection distance, with longer distances achieved later in the day. Among meteorological factors, besides major positive effect of humidity, temperature and wind had a negative effect, although the estimated effect sizes were minor. Best models’ results are listed in Supplementary Table 2.

### Differences between pine and beech forest

**Figure 2:**
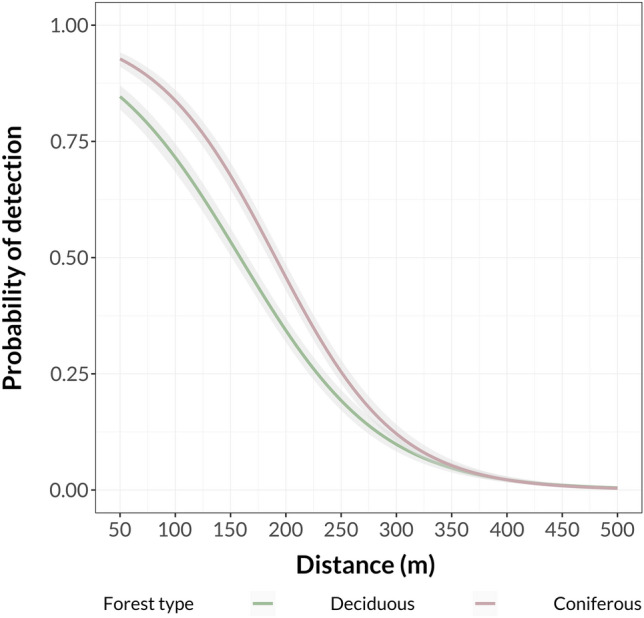
Fitted detection probability curves for all species at both transects. Curves were fitted with the generalized linear model method with a 95% confidence interval showed as a shaded area.

In general, the detection probabilities of vocalisations were significantly lower (p = 0.022) in the deciduous forest than in the coniferous one (754 vocalisation detected (45% of all detections) vs. 938 (55%) (Fig. 2). Modelling of maximum detection distances revealed significantly greater distances in the coniferous forest (p < 0.001).

### Season effect

Of all vocalisations in the coniferous and deciduous forests, 18.6% and 14.0%, respectively, were detected in April, while 18.6% and 17.3% were detected in May and 18.3% and 13.2% in June. The detection rate between months was very consistent at the coniferous site and varied in the deciduous transect.

**Figure 3:**
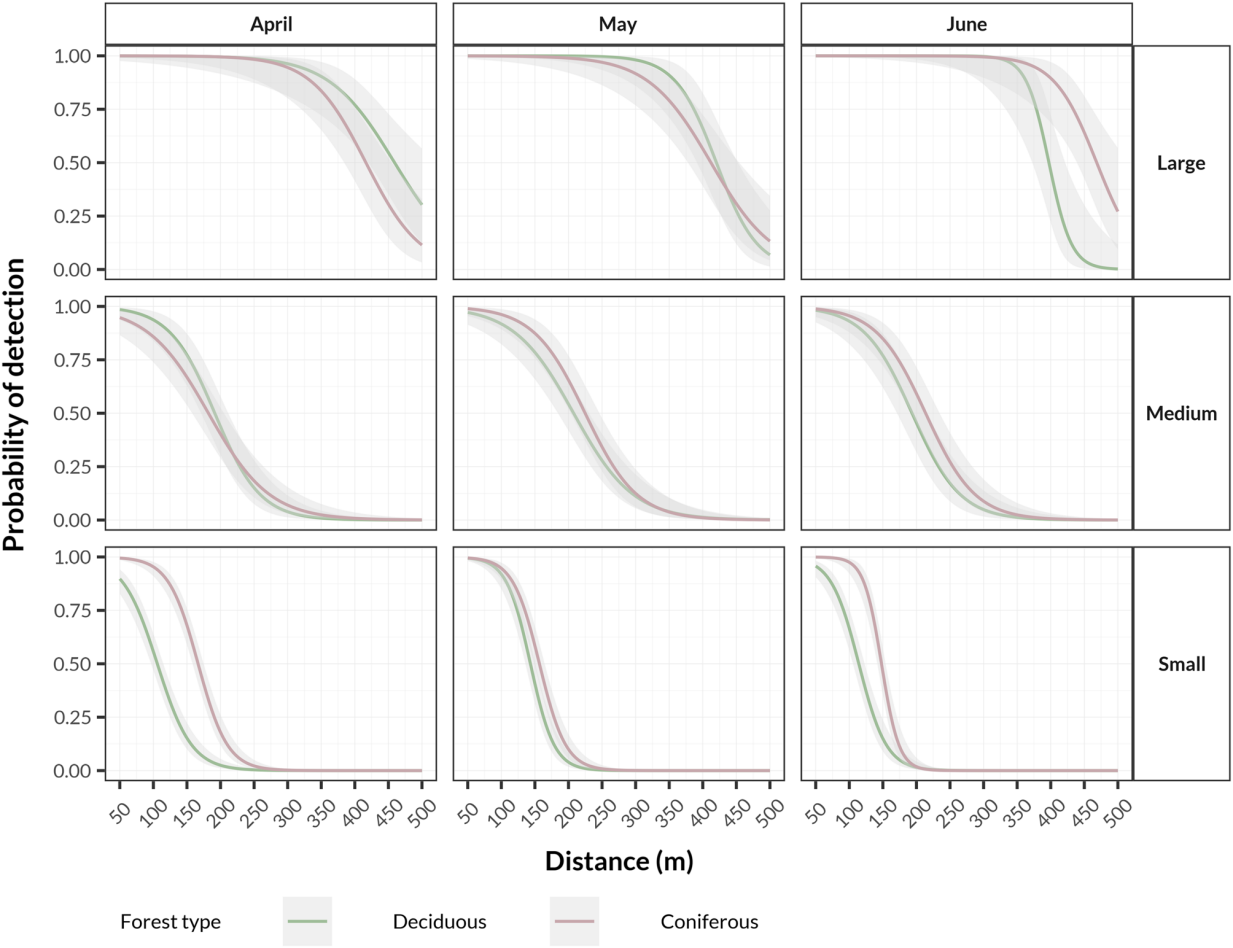
Changes in detection probability during the season in both habitats for species divided into size-related groups based on the principal component analysis. Curves were fitted with the generalized linear model method with a 95% confidence interval showed as a shaded area.

Throughout the season, the general detection probability was higher in coniferous forests for all groups of birds, with the exception of large species in April and May (Fig. 3).

### Effect of singing height

In both transects, least detections were achieved at 9 m height. Also, in both environments there were only minor differences between 3 and 6 m (34.2% vs. 34.7%, respectively).

**Figure 4:**
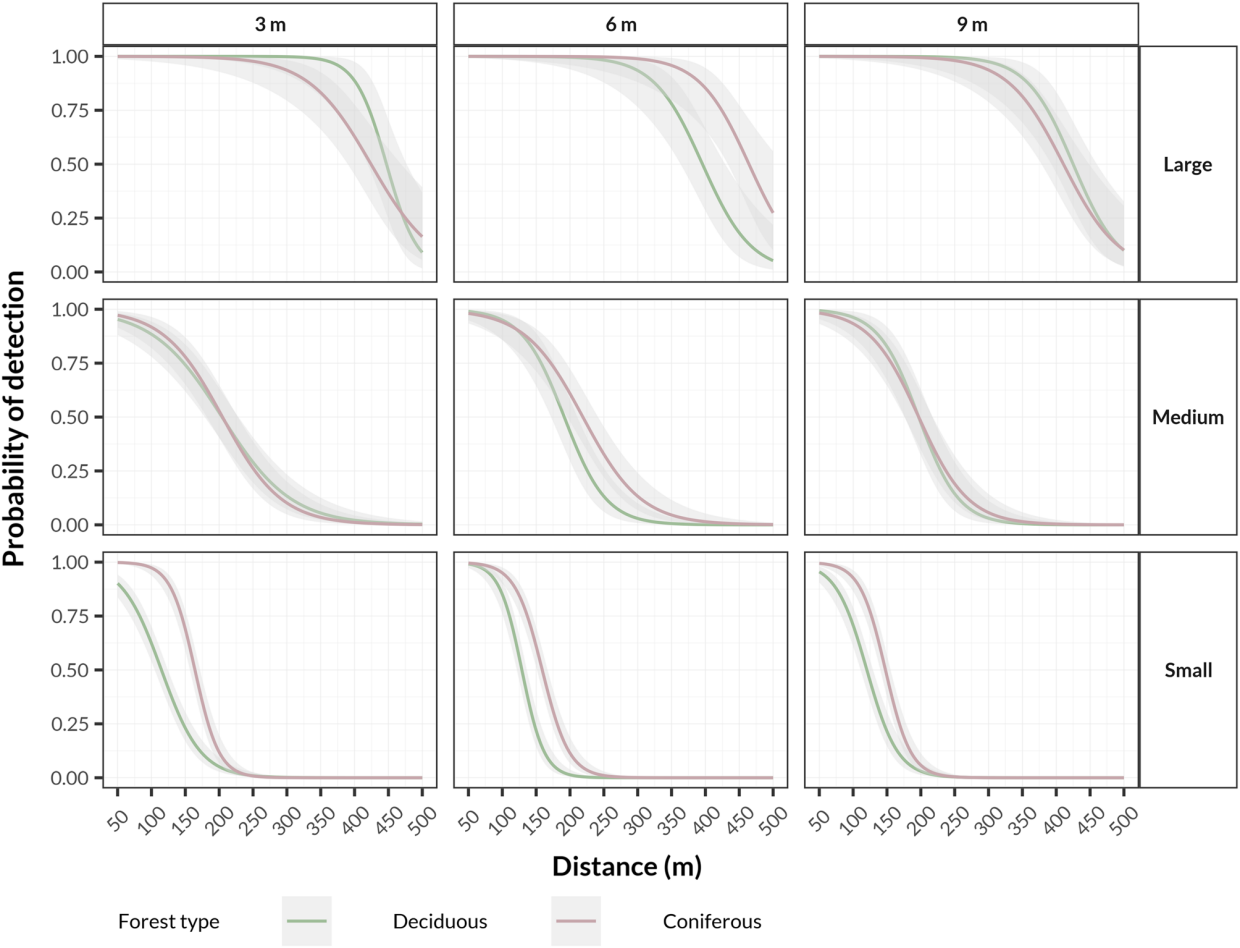
Changes in detection probability in both habitats for species divided into size-related groups based on the principal component analysis according to broadcasting height. Curves were fitted with the generalized linear model method with a 95% confidence interval showed as a shaded area.

For each group of birds, the probability of detection was higher in the coniferous forest at almost any height, again with the exception of large birds (Fig. 4). Changes in detection probability for different broadcasting heights during the season can be seen in Supplementary Fig. 1.

### Species effect

**Figure 5:**
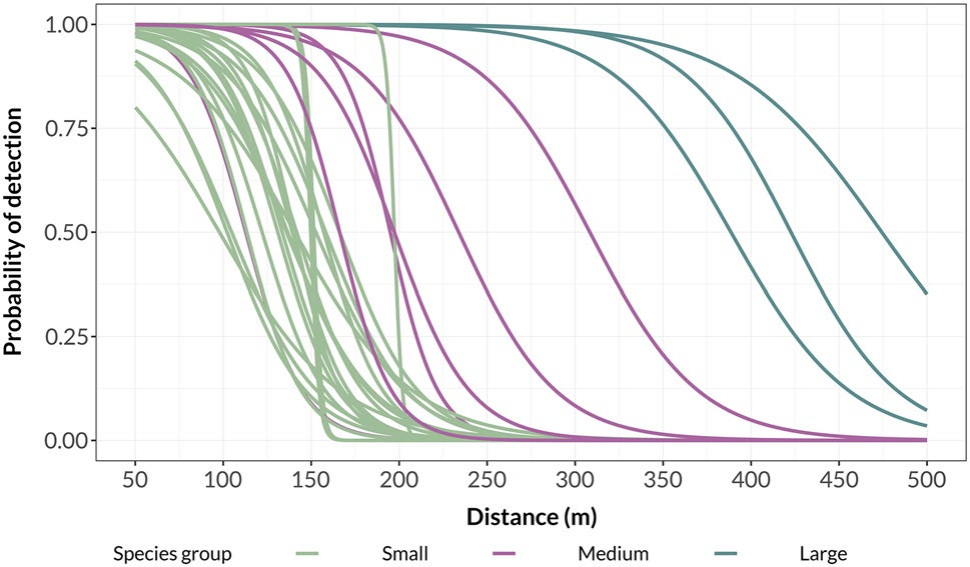
Predicted probability of detection of each species at different distances from the speaker. Curves were fitted with generalized linear model method. Each species assigned to a size group based on the principal component analysis is showed as a separate curve.

The effects of SPL and peak frequency were consistent across species. For some smaller species, the detection probability dropped rapidly at a certain distance (Fig. 5). The species with the highest probability of detection was the common cuckoo *Cuculus canorus*, while the species with the lowest was the long-tailed tit *Aegithalos caudatus*.

The common cuckoo and stock dove *Columba oenas* were the only two species whose vocalisations were detected at a distance of 500 m. Vocalisations of most of the studied species were usually not visually detectable on spectrograms at a distance of 150 m, although they were still audible on recordings even at 200 m. Table 3 presents the detection probability for all species based on logistic regression. The measurements incorporate all of the observations from this study, and in many cases they are shorter than the extreme distances we noted in exceptional cases during the study. Detection probability for each species can be seen in the Supplementary Fig. 2.

**Table 3:** Distances (extracted from detection models) at which each species could be detected with an assumed probability between 0.05 and 0.95.

	0.95	0.90	0.80	0.50	0.20	0.10	0.05	Deciduous Territory Radius (m)	Coniferous Territory Radius (m)
*Columba palumbus*	290	315	342	389	435	462	488	65–123	80–157
*Dryocopus martius*	217	240	266	308	351	376	399	252–564	178–399
*Columba oenas*	333	356	381	423	465	489	512		95–230
*Accipiter nisus*	155	165	176	195	214	225	235		
*Cuculus canorus*	351	382	416	474	532	567	598	146–326	113–230
*Turdus philomelos*	134	150	167	197	227	244	260	50–88	52–94
*Turdus merula*	123	134	146	166	187	199	210	53–95	65–118
*Dendrocopos major*	66	78	91	113	135	148	160	63–133	67–113
*Loxia curvirostra*	82	95	109	134	158	172	185		113–399
*Oriolus oriolus*	152	173	195	234	272	295	315	146–564	103–399
*Anthus trivialis*	69	82	97	122	147	162	175		178–^†^
*Lullula arborea*	100	117	135	166	197	215	232		
*Parus major*	190	192	194	197	200	202	204	45–86	55–105
*Sylvia atricapilla*	86	100	115	142	168	183	198	40–74	52–95
*Luscinia megarhynchos*	90	108	126	158	190	209	226		
*Fringilla coelebs*	90	108	126	158	190	209	226	26–44	32–55
*Prunella modularis*	37	53	71	102	133	151	168	178–^†^	84–188
*Erithacus rubecula*	42	67	94	141	187	214	239	38–71	40–72
*Ficedula hypoleuca*	33	51	71	105	138	158	176	126–326	
*Lophophanes cristatus*	108	120	134	157	180	194	207	126–^†^	80–151
*Cyanistes caeruleus*	144	146	148	151	155	157	158	56–107	89–252
*Troglodytes troglodytes*	78	97	117	153	188	208	227	61–111	113–^†^
*Ficedula parva*	64	82	102	136	170	189	208	113–^†^	178–^†^
*Phylloscopus trochilus*	101	111	122	141	159	170	180		126–^†^
*Poecile palustris*	81	94	107	131	155	169	181	80–146	126–^†^
*Certhia brachydactyla*	92	104	117	139	161	174	186		
*Certhia familiaris*	143	145	147	150	153	155	157	89–151	84–163
*Phylloscopus sibilatrix*	143	144	146	150	153	155	157	36–163	49–97
*Aegithalos caudatus*	*	22	50	98	146	174	199	252	178–^†^
*Phylloscopus collybita*	69	86	105	137	169	187	205	113–^†^	45–87
*Regulus regulus*	68	79	92	113	135	148	159	89–200	76–146

For species with lower detectability, the highest probability value goes to zero (*). Territory radii are based on density measures from Bialowieza primaeval forest and represent the minimum and maximum range obtained over several years of study^18^. Maximum ranges for small specialised species are inflated due to the probable heterogeneity of the habitat (^†^).

## Discussion

In this study, we estimated the maximum detection distances that could be obtained from sound propagation experiments using territorial vocalisations of 31 European bird species. We studied the effect of physical properties of the songs/calls, weather, and habitat variables on the detection probability at different distances from a speaker. Our study represents a move towards the standardisation of acoustic surveys, which is important for planning research or monitoring using the PAM approach.

### Species

Our results revealed that body mass and SPL are the best predictors of detection probability. The higher the SPL and the larger the species, the longer the distance at which the sound can be heard. According to Brenowitz, only 1/3 of the variation in SPL is attributable to changes in body weight. Therefore, the simplistic assumption that body mass can predict SPL—and thus detection distance—is incorrect, and such approximations must be performed with caution. There are indeed species of similar body mass that relevantly differ in detection probability, such as the common cuckoo and Eurasian sparrowhawk *Accipiter nisus*. Hence, vocal parameters and body mass should be considered collectively when evaluating detection distance. However, song frequency can, to a certain degree, be predicted by body mass^24^ and this information could be useful in estimating characteristics of lesser-known species for the purpose of research planning. This factor is highly species-specific, as forest birds generally produce more pure tones, and the frequency of their songs is less variable than in species living in other habitats^25^. In the forest, the sound frequency for optimal communication is around 1.6–2.5 kHz^25^, because higher frequencies attenuate at shorter distances^26,27^. Other effects, such as reverberation, also predominantly influence high frequencies (above 8 kHz), while frequencies below 2 kHz are less affected^28,10^. Most of the small bird species in our study are characterised by a frequency range oscillating from about 1500 to 8500 Hz, which translates to a maximum detection distance of 150 m. Instead, species characterised by the 100–2500 Hz frequency range were detected at much further distances. This type of information is useful for planning PAM surveys based on the species of interest, as it can be used to set expectations for the detectability and maximum distance possible in optimal environmental conditions. Knowing at least one of the vocal parameters might be helpful in those estimations.

Even though the height effect was substantial for both detection probability and maximum distance, the overall effect sizes were low (Table 2). In terms of PAM planning, the height of singing is not something that can be set by the researcher. Different species prefer different song posts, which are determined only by the opportunities given by the habitat structure. However, knowledge about a species' singing behaviour and environmental characteristics should help in the decision-making stage and in the proper interpretation of the data obtained. For example, the monitoring of low-amplitude species that sing from higher positions above the ground will require the deployment of ARUs at higher densities.

### Type of forest

We found that forest type had a major effect on the maximum detection distance and detection probability. Species were detected at farther distances in the coniferous forest than in the deciduous, and more detections were recorded there (55.4% vs. 44.6%). Under differing conditions, the same acoustic signal can experience different distortions; in a forest habitat, the most important types of distortions are amplitude fluctuations and reverberation, the strength of which depends on the forest type^28,10^. The coniferous forest in our study is characterised by a higher homogeneity of smaller-crowned, more densely packed trees than the deciduous forest, which might be one of the reasons for the consistent difference between the two transects. However, as sound propagation cannot be predicted based on vegetation structure^9^ and forest types differ in terms of structural properties, the type of forest is only a minor consideration in PAM planning—researchers are much more likely to choose an area of interest based on the species that inhabit it rather than its inherently higher or lower probability of detection.

### Limitations

**Figure 6:**
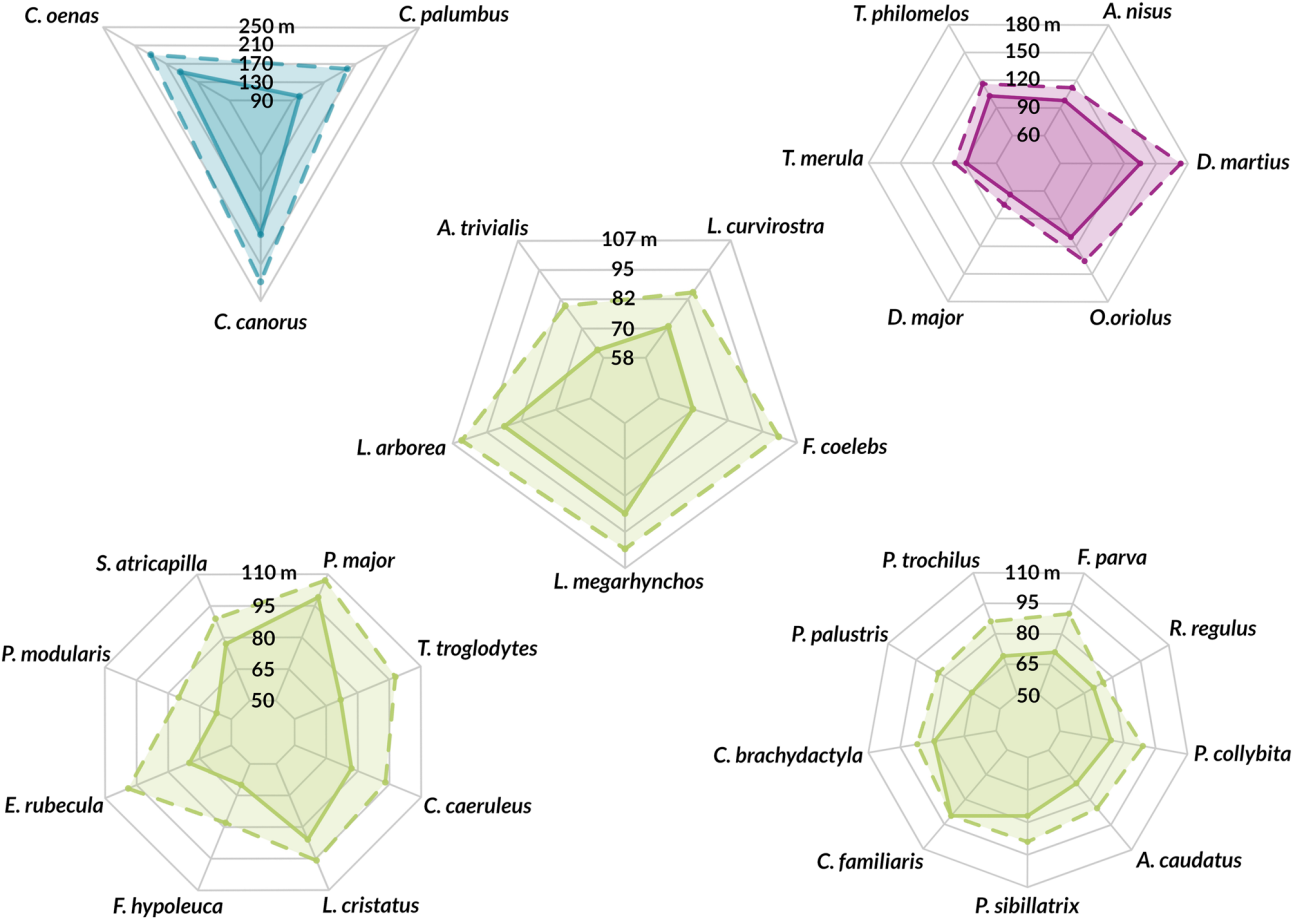
Comparison of species detection distances. The dashed line is the mean distance (m) at which the species could be heard, while the solid line is the mean distance (m) at which species could be detected visually on a spectrogram.

In this study, we were able to determine ranges for audible and spectrogram-visible detections (Fig. 6) that should be considered when planning an analysis of recordings. Depending on the type of detection intended (automatic or manual), such differences in detectability can be an important factor in PAM planning. At a distance of 150 m, we could hear all 31 species, but only 64.52% of them were visible on the spectrogram. Vocalisations of most small birds were still visible at 100 m, and for large species this threshold was about 300–400 m, although they could be heard at much greater distances. This difference is the result of two factors: the effective range of the recorder, which gathered distant sounds that were not loud enough to be visually represented, and masking by other birds, which was likely high in this study due to the short duration (1 min) of the recordings. Masking should not be as much of a problem in long-term acoustic monitoring as there is more likely to eventually be a sound window for the species of interest to fit in. However, in our study, the time regime was kept deliberately short to facilitate analysis. Here, the greatest difference in the maximum distance of visual and audio detection was obtained for the European robin, *Erithacus rubecula* (29%). This species was frequently masked in audio recordings from greater distances and its vocalisations were difficult to detect visually. Instead, the mean value for all species was 16%.

Researchers should keep in mind that the estimations presented in our study will vary (most likely be shorter) depending on the habitat in question^29^. Depending on the time of day and environmental factors that influence sound propagation, the maximum communication distance of a bird song can vary by about several dozen metres throughout the day^24^.

For more detailed guides, species could be organised into groups of families or genera according to the similarity of their vocalisations, since certain call traits, such as frequency, determine the range at which they can be detected^11^. This methodology could potentially be used for the species whose detection distances have not yet been estimated. If a species of interest is not listed in this work, we suggest comparing its sound parameters with a species from this study that is close to its vocal values.

### Recommendations

We suggest the following simple guidelines for planning research with the use of ARUs. If the research is focused on a single species, we recommend measuring its song amplitude directly and performing a field test similar to the one presented here. This will greatly facilitate later interpretation of the data. For more comprehensive studies, we developed the following advice for obtaining the most effective results.

PAM planning will depend on researcher’s approach. Either individual should not be recorded on two devices at the same time, and it can be treated as a spatial point within the recorder’s range, or the individual can be recorded on multiple ARUs while singing from different points at different time, and it should be treated as a point cloud. The second approach will result in deploying the recorders closer together, as varied detectability on each device can provide additional data, e.g. on spatial distribution.

If the species of interest is listed in this study, we propose using Table 3 as a reference to estimate the placement of ARUs. To properly determine the minimum distance between ARUs, researchers should choose a maximum detectability range adjusted to the potential territory size and territory spacing of the chosen species. Decisions about the monitoring setup will then reflect the goals at hand. A first consideration in planning where to deploy ARUs is the size and density of the bird population, as having devices in close proximity might result in recordings of the same individual on several ARUs. For studies of species that possess large home ranges, though, sparsely distributed ARUs may not generate results if a bird of interest will not call or sing in their proximity, away from its central territory. However, this approach could still provide information on whether the species is present there. With a limited number of devices, the decision on where to deploy ARUs will reflect a trade-off between the size of the potential area of a species’ occurrence and the area that can be effectively covered by the devices.

**Figure 7:**
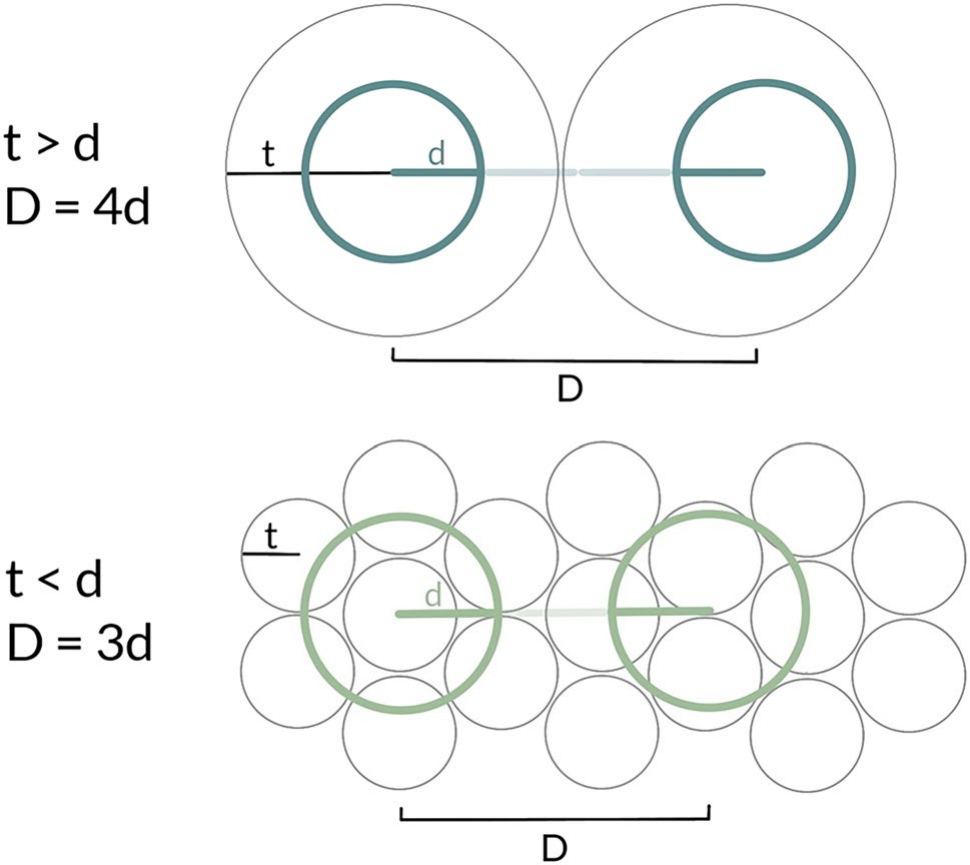
PAM planning formula based on the size of a species’ territory and its detection range. Grey circles are species’ home ranges with radii (t); coloured circles show detection ranges with radii (d), with the recorders placed at their centres. The distance between recorders is marked as D.

Regarding this point, we have developed a simple formula based on territory size and detectability range to follow while planning PAM for various species (Fig. 7). For research on birds whose territories are smaller than their maximum detectability range, we propose separating recorders with a distance equal to triple the range (3d). This type of monitoring scheme would provide recordings of separate individuals in close proximity to the recorder, while possibly gathering some distant sounds of other individuals as well, particularly if their home ranges partially overlap or are close by. As large species with larger detection ranges often occupy large territories, deciding where to deploy devices will depend on whether the species is territorial and the size of its territories. For birds with a territory larger than their detection range, we propose quadrupling the detection distance for the placement of ARUs (4d). This would ensure the independence of data for territorial birds that occupy areas of, e.g., double the size of their detectability. For those that move throughout very large distances, such as the common cuckoo, it increases the probability that the bird will be recorded on any device. The estimated distance can be adjusted according to a species’ potential range and vocal characteristics.

In Table 3 we present mean radii of detectability and territory size for the species examined in this study. However, the home ranges of certain species might vary as some inhabit large areas or are not territorial. For example, the common cuckoo travels a distance of several kilometres throughout the day while the wood pigeon *Columba palumbus* may defend only the area in close proximity to its nest. Therefore, PAM of species that are difficult to assess in terms of their presence and area usage will need to be flexible and adapted to a given situation and its research needs.

For any species not included here, as well as those that are, researchers should gather information about the frequency and amplitude at which the species of interest is vocalising and, based on that information, estimate its detection radius. Because birds differ in vocal characteristics both between and within species, a reasonable range should be determined based on the broadcast signals (usually songs). This estimate should be compared to the potential size of the species’ territory to determine how much space will need to be covered with ARUs in order to collect information about all individuals in the area.

Finally, the area of interest should also be analysed, as its structure, vegetation cover, and topography may influence the assessed distances in both positive and negative ways. The present study was conducted in neutral, flat terrain; in hillier or more variable terrain we suggest reducing the estimated detection radius to ensure that any negative impact on detection will be mitigated.

### Supplementary Information

Supplementary Information.

## Data Availability

The recordings and datasets analysed during the current study are available from the corresponding author on reasonable request (Dominika Winiarska, email: dmwiniarska1@gmail.com).
