# Supplementary material for: Detection ranges of forest bird vocalisations: guidelines for passive acoustic monitoring
Source: Sci Rep. 2024 Jan 9;14:894. doi: 10.1038/s41598-024-51297-z (PMC10776575; doi:10.1038/s41598-024-51297-z)
Supplement: Supplementary file 1 — Supplementary Information. [file 41598_2024_51297_MOESM1_ESM.pdf]

Detection ranges of forest bird vocalisations: guidelines for passive acoustic monitoring

Authors Dominika Winiarska[1]*, Paweł Szymański[1], Tomasz S. Osiejuk[1]

[1] Department of Behavioural Ecology, Institute of Environmental Biology, Faculty of Biology, Adam Mickiewicz University, Poznań, Poland

* dominika.winiarska@amu.edu.pl

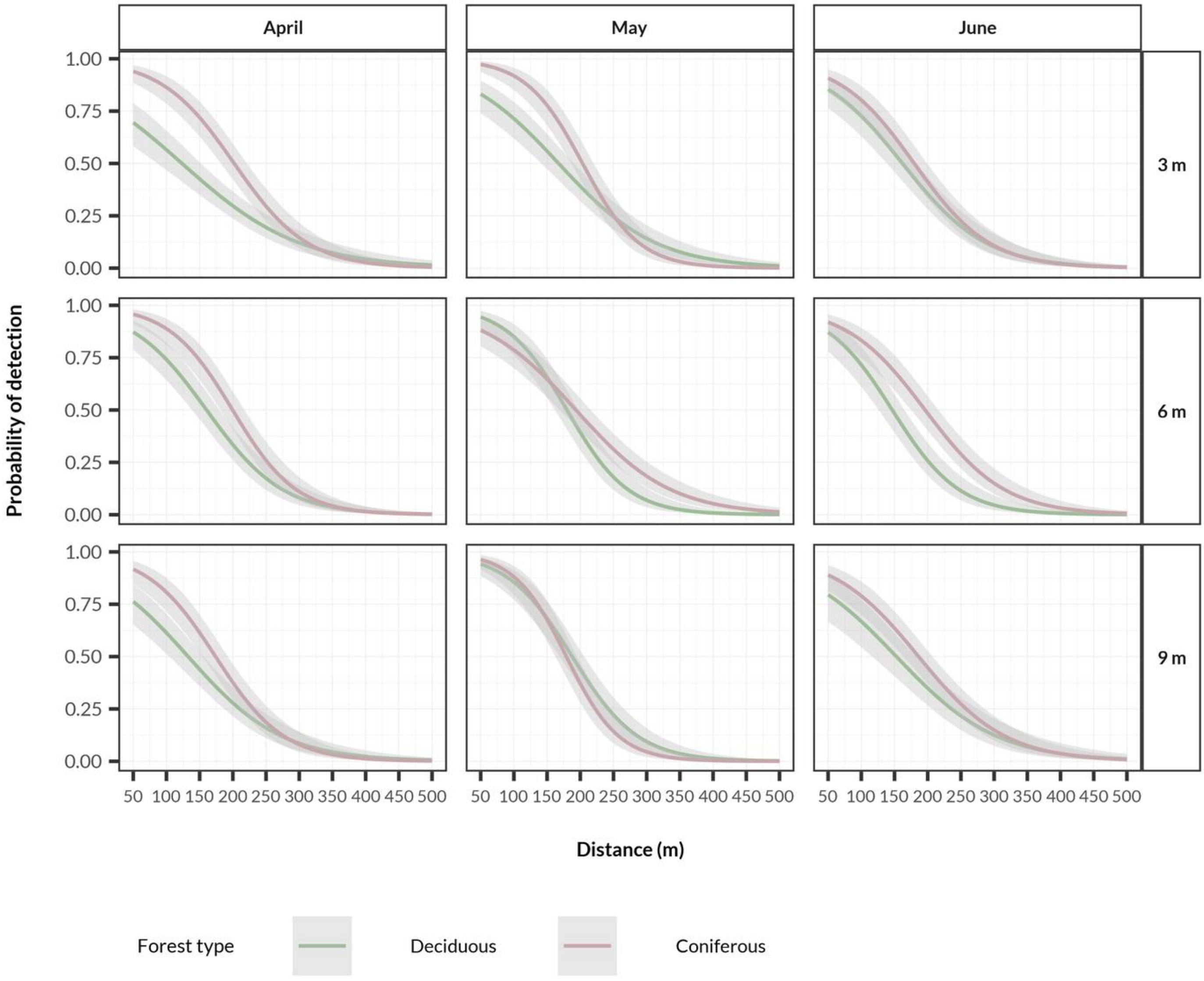

Supplementary Figure 1. Changes in detection probability for different broadcasting heights during the season. Curves were fitted with generalized linear model method with 95% confidence interval showed as a shaded area.

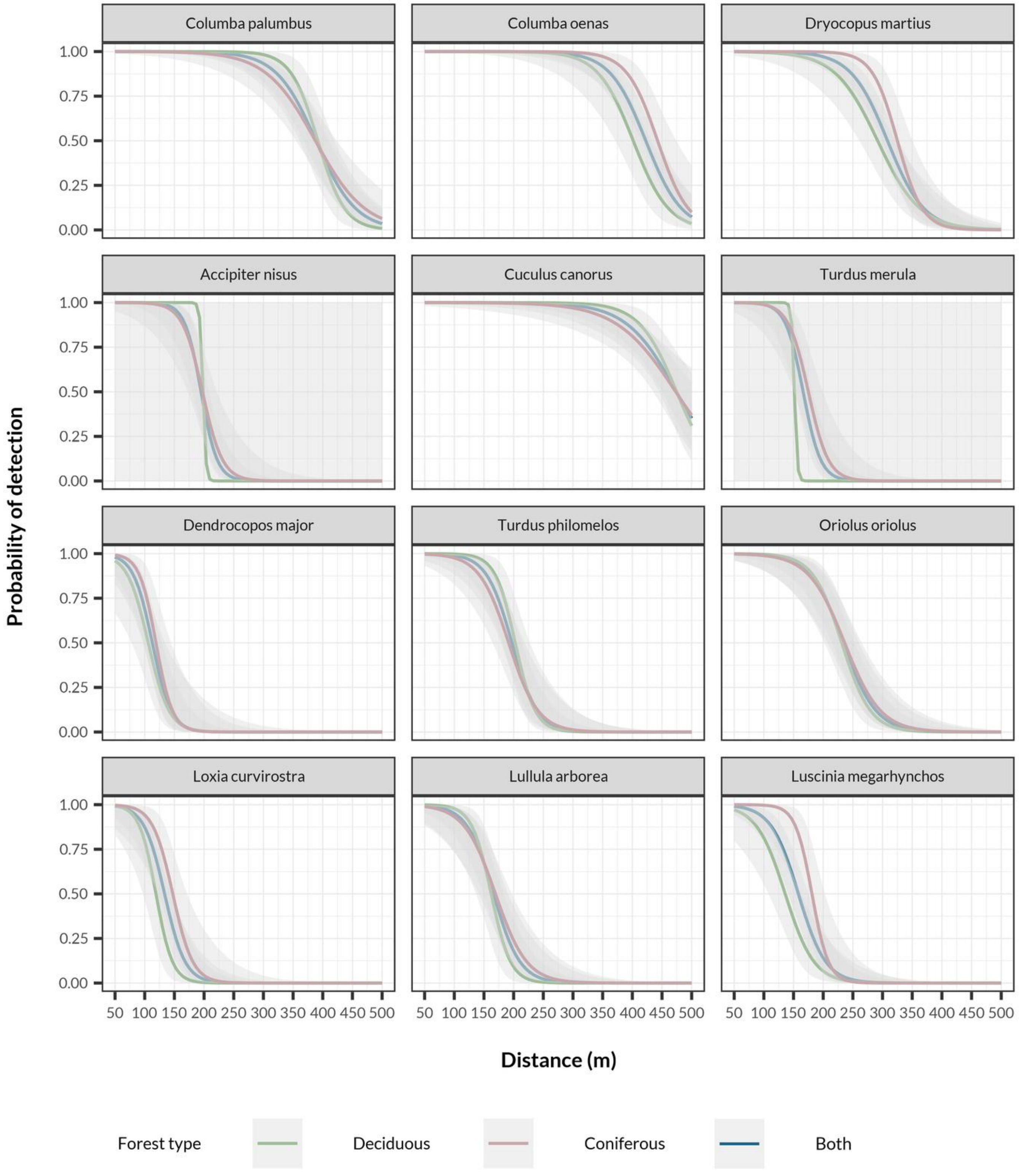

Supplementary Figure 2a. General detection probability of each species in each habitat. Curves were fitted with generalized linear model method with 95% confidence interval showed as a shaded area.

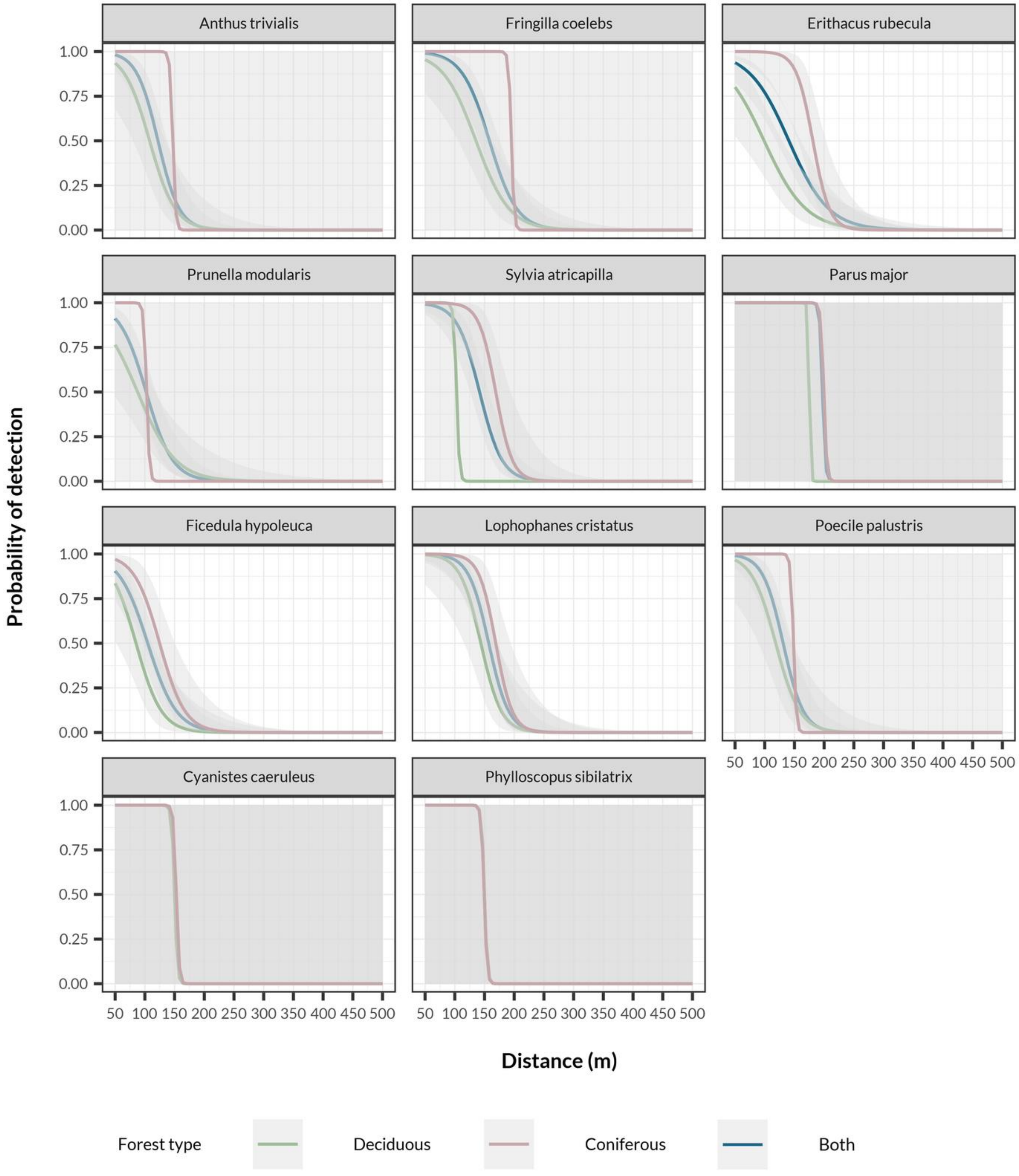

Supplementary Figure 2b. General detection probability of each species in each habitat. Curves were fitted with generalized linear model method with 95% confidence interval showed as a shaded area.

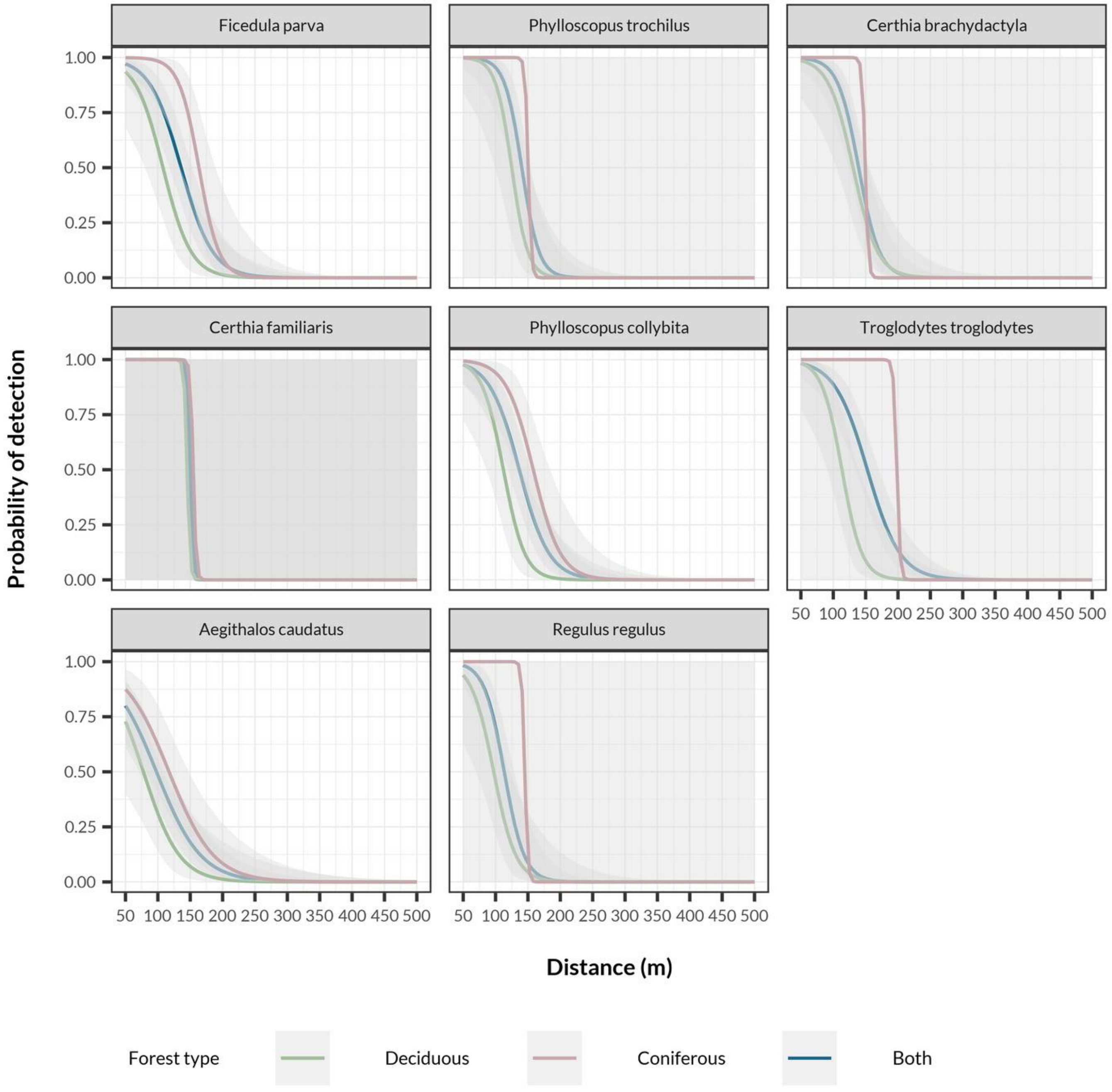

Supplementary Figure 2c. General detection probability of each species in each habitat. Curves were fitted with generalized linear model method with 95% confidence interval showed as a shaded area.

Supplementary Table 1. Meteorological data on each experiment setup.

Transect	Date	Time (am)	Broadcast height	Wind (m/s)	Humidity (%)	Temperature (°C)
Coniferous	01.04.2021	07:05	3 m	0,00	75,8	7,2
		07:50	9 m	0,00	82,1	8,1
		08:30	6 m	0,00	80,2	10,3
Deciduous	02.04.2021	07:05	9 m	0,22	74,1	0,8
		07:50	3 m	0,22	79,5	2,0
		08:40	6 m	0,00	69,3	3,9
Coniferous	12.05.2021	06:05	6 m	0,00	74,3	14,1
		06:50	3 m	0,00	75,2	14,8
		07:32	9 m	0,36	67,5	17,1
Deciduous	14.05.2021	06:05	3 m	0,62	80,7	12,0
		06:53	6 m	0,52	83,7	12,0
		07:36	9 m	0,29	83,5	12,2
Coniferous	08.06.2021	05:50	9 m	0,00	79,2	11,3
		06:35	6 m	0,00	78,8	12,8
		07:20	3 m	0,00	76,5	14,9
Deciduous	09.06.2021	05:58	9 m	0,47	66,4	15,5
		07:23	3 m	0,59	62,6	16,6
		08:06	6 m	0,75	59,9	17,3

Supplementary Table 2. Best models of detection probability and maximum distance used for the analysis based on Akaike's information criterion (AIC).

Detection ~ Distance + Transect + Height + Month + Body mass + SPL + Time of day + Temperature + Humidity + Wind + (1 + Distance | Species)					
	df	Log-likelihood	AICc	Delta	Weight
	14	-758.025	1544.1	0.00	0.160
		Estimate	Std. Error	z value	p value
	Intercept	2.901	0.453	6.409	<0.001
	Distance	-1.497	0.101	-14.792	<0.001
	Month	-0.183	0.079	-2.319	0.020
	Transect Beech	-0.305	0.135	-2.256	0.024
	Height	-0.132	0.048	-2.744	0.006
	Time of day	-0.353	0.182	-1.943	0.052
	Humidity	0.639	0.141	4.527	<0.001
	Temperature	1.328	0.269	4.931	<0.001
	Wind	-0.568	0.204	-2.777	0.005
	SPL	2.582	0.658	3.927	<0.001
	Body mass	4.722	0.840	5.619	<0.001
Detection ~ Distance + Transect + Height + Month + Body mass + Peak frequency + SPL + Time of day + Temperature + Humidity + Wind + (1 + Distance | Species)					
	df	Log-likelihood	AICc	Delta	Weight
	15	-757.349	1544.8	0.66	0.115
		Estimate	Std. Error	z value	p value
	Intercept	3.789	0.894	4.236	<0.001
	Distance	-1.504	0.104	-14.474	<0.001
	Month	-0.183	0.079	-2.310	0.021
	Transect Beech	-0.303	0.135	-2.247	0.025
	Height	-0.132	0.048	-2.746	0.006
	Time of day	-0.356	0.182	-1.957	0.050
	Humidity	0.641	0.141	4.531	<0.001
	Temperature	1.329	0.269	4.935	<0.001
	Wind	-0.569	0.205	-2.782	0.005
	Peak frequency	-1.227	1.058	-1.160	0.246
	SPL	2.271	0.691	3.286	0.001
	Body mass	4.112	0.963	4.268	<0.001
Detection ~ Distance + Transect + Height + Month + Body mass + SPL + Call duration + Time of day + Temperature + Humidity + Wind + (1 + Distance | Species)					

df	Log-likelihood	AICc	Delta	Weight
15	-757.373	1544.8	0.71	0.112
	Estimate	Std. Error	z value	p value
Intercept	2.762	0.465	5.937	<0.001
Distance	-1.497	0.101	-14.804	<0.001
Month	-0.183	0.079	-2.315	0.021
Transect Beech	-0.305	0.135	-2.259	0.024
Height	-0.132	0.048	-2.737	0.006
Time of day	-0.353	0.182	-1.939	0.053
Humidity	0.639	0.141	4.523	<0.001
Temperature	1.326	0.269	4.925	<0.001
Wind	-0.567	0.204	-2.774	0.006
SPL	2.365	0.669	3.534	<0.001
Body mass	4.405	0.874	5.040	<0.001
Call duration	0.781	0.679	1.151	0.250
Detection ~ Distance + Transect + Height + Month + Body mass + Peak frequency + SPL + Call duration + Time of day + Temperature + Humidity + Wind + (1 + Distance | Species)				
df	Log-likelihood	AICc	Delta	Weight
16	-756.806	1545.7	1.58	0.072
	Estimate	Std. Error	z value	p value
Intercept	3.580	0.905	3.954	<0.001
Distance	-1.504	0.104	-14.500	<0.001
Month	-0.182	0.079	-2.308	0.021
Transect Beech	-0.304	0.135	-2.250	0.024
Height	-0.132	0.048	-2.740	0.006
Time of day	-0.355	0.182	-1.952	0.051
Humidity	0.640	0.141	4.527	<0.001
Temperature	1.328	0.269	4.929	<0.001
Wind	-0.568	0.205	-2.779	0.005
Peak frequency	-1.111	1.049	-1.059	0.290
SPL	2.108	0.696	3.026	0.002
Body mass	3.882	0.981	3.959	<0.001
Call duration	0.702	0.671	1.047	0.295
Detection ~ Distance + Transect + Height + Month + Body mass + SPL + Temperature + Humidity + Wind + (1 + Distance | Species)				
df	Log-likelihood	AICc	Delta	Weight

13	-759.919	1545.9	1.78	0.066
	Estimate	Std Error	Z value	P value
Intercept	2.926	0.450	6.498	<0.001
Distance	-1.493	0.101	-14.808	<0.001
Month	-0.201	0.078	-2.560	0.010
Transect Beech	-0.355	0.132	-2.679	0.007
Height	-0.131	0.048	-2.726	0.006
Humidity	0.604	0.140	4.320	<0.001
Temperature	1.170	0.256	4.568	<0.001
Wind	-0.572	0.204	-2.801	0.005
SPL	2.568	0.655	3.922	<0.001
Body mass	4.714	0.838	5.626	<0.001
Max distance ~ Transect + Height + Month + Body mass + Peak frequency + SPL + Time of day + Temperature + Humidity + (1 | Species)				
df	Log-likelihood	AICc	Delta	Weight
12	-607.7	1239.3	0.0	0.48
	Estimate	Std. Error	z value	p value
(Intercept)	-2.514	1.261	-1.993	0.046
Transect Pine	0.118	0.023	5.080	<0.001
Height	-0.041	0.013	-3.179	0.001
Month	-0.047	0.020	-2.381	0.017
Time of day	-0.073	0.035	-2.067	0.039
Humidity	0.517	0.129	4.001	<0.001
Temperature	0.109	0.045	2.433	0.015
Body mass	0.184	0.056	3.295	0.001
Peak frequency	-0.361	0.147	-2.453	0.014
SPL	3.360	1.178	2.851	0.004
Max distance ~ Transect + Height + Month + Body mass + Peak frequency + SPL + Call duration + Time of day + Temperature + Humidity + (1 | Species)				
df	Log-likelihood	AICc	Delta	Weight
13	-607.1	1240.3	1.0	0.28
	Estimate	Std. Error	z value	p value
(Intercept)	-2.349	1.251	-1.878	0.060
Transect Pine	0.118	0.023	5.065	<0.001
Height	-0.041	0.013	-3.178	0.001
Month	-0.047	0.020	-2.375	0.018

Time of day	-0.072	0.035	-2.06	0.039
Humidity	0.517	0.129	4.005	<0.001
Temperature	0.109	0.045	2.435	0.015
Call duration	0.099	0.096	1.034	0.301
Body mass	0.165	0.058	2.852	0.004
Peak frequency	-0.340	0.146	-2.324	0.020
SPL	3.097	1.187	2.61	0.009

Max distance ~ Transect + Height + Month + Body mass + Peak frequency + SPL + Time of day + Temperature + Humidity + Wind + (1 \| Species)

df	Log-likelihood	AICc	Delta	Weight
13	-607.3	1240.6	1.4	0.24

	Estimate	Std. Error	z value	p value
(Intercept)	-2.519	1.262	-1.997	0.046
Transect Pine	0.095	0.036	2.65	0.008
Height	-0.043	0.013	-3.291	0.001
Month	-0.050	0.020	-2.496	0.013
Time of day	-0.073	0.035	-2.081	0.037
Humidity	0.532	0.130	4.094	<0.001
Temperature	0.130	0.052	2.523	0.012
Wind	-0.045	0.054	-0.839	0.401
Body mass	0.184	0.056	3.299	0.001
Peak frequency	-0.360	0.147	-2.444	0.015
SPL	3.364	1.179	2.853	0.004
